# Asymmetric cell division via DNA strand-specific epigenetic imprinting and segregation explains eukaryotic development

**DOI:** 10.1186/1756-8935-6-S1-P115

**Published:** 2013-04-08

**Authors:** Amar JS Klar

**Affiliations:** 1 Developmental Genetics Section, Gene Regulation and Chromosome Biology Laboratory, National Institutes of Health, Frederick National Laboratory for Cancer Research, Frederick, MD, USA

## 

The asymmetric cell division is crucial during development and to produce self-renewing stem cells. A large body of work on *Drosophila* and *C. elegans* suggests that this process occurs by non-equivalent distribution of proteins and/or mRNA (intrinsic factors) to daughter cells, or by their differential exposure to cell extrinsic factors. In contrast, strictly based on template “Watson” versus “Crick” strand inherited from the parental cell, haploid fission yeast sister cells developmentally differ by inheriting sister chromatids that are differentiated by epigenetic means. Specifically, the double helical structure of DNA is the ultimate determinant of asymmetric cell division. To employ this kind of mechanism for cellular differentiation in diploid organisms, selective segregation must occur for partitioning epigenetically differentiated sister chromatids from both chomosome homologs to specific daughter cells, at a specific cell division, during multicelluar organism development. We previously proposed that Somatic Sister chromatid Imprinting and Selective chromatid Segregation (*SSIS*) model might explain development in eukaryotes, such as that of the body left-right axis laterality specification in mice and brain laterality in humans. However, for technical reasons, it is impossible to determine whether such a mechanism operates during embryonic development in higher organisms.

Recently we discovered the second example of DNA strand-specific imprinting mechanism in *Schizosaccharomyces japonicus* yeast (36% GC content), whose DNA sequence is only about 30% similar compared to the well-studied *S. pombe* (44% GC). Therefore these highly diverged yeasts provide the first two examples in which the intrinsic chirality of double helical structure of DNA forms the primary determinant of asymmetric cell division [1]. I will also discuss support for our model to explain body laterality development in mice [2]. Interestingly, another study also suggested that *C. elegans* neuronal asymmetry develops via the SSIS model [3].
